# A Green Protocol for Microwave-Assisted Extraction of Volatile Oil Terpenes from *Pterodon emarginatus* Vogel. (Fabaceae)

**DOI:** 10.3390/molecules23030651

**Published:** 2018-03-13

**Authors:** Giuliana M. Vila Verde, Diogo A. Barros, Marilene Silva Oliveira, Gilberto L.B. Aquino, Danilo M. Santos, José Realino de Paula, Lucas D. Dias, Marta Piñeiro, Mariette M. Pereira

**Affiliations:** 1 Laboratório de Bioprodutos e Síntese, Campus Anápolis de Ciências Exatas e Tecnológicas-Henrique Santillo, Universidade Estadual de Goiás, BR 153 nº 3.105, Fazenda Barreiro do Meio, Anápolis 75132-903, Goiás, Brazil; amorimbarrosadvocacia@gmail (D.A.B.); marilenes36@gmail.com (M.S.O.); gilberto.benedito@ueg.br (G.L.B.A.); 2 Instituto de Química de São Carlos, Universidade de São Paulo, Av. Trab. São-Carlense, 400-Parque Arnold Schimidt, São Carlos-SP, São Carlos 13566-590, Brazil; danilo_martins@usp.br; 3 Laboratório de Pesquisa em Produtos Naturais, Faculdade de Farmácia, Universidade Federal de Goiás, Av. Universitária com 1^a^ Avenida s/n, Setor Universitário, Goiânia 74605-220, Goiás, Brazil; pjrpaula@gmail.com; 4 CQC, Departamento de Química, Universidade de Coimbra, Rua Larga, 3004-535 Coimbra, Portugal; lucasdanillodias@gmail.com (L.D.D.); pineiromarta@gmail.com (M.P.); mmpereira@qui.uc.pt (M.M.P.)

**Keywords:** optimisation, microwave, volatile oil, extraction, terpenes, *Pterodon emarginatus*, analytical eco-scale

## Abstract

Microwave-assisted extraction of volatile oils (MAE) potentially offers a more efficient and bio-sustainable method than conventional extraction by Clevenger apparatus (CE). This study aimed to optimise the MAE of the volatile oil from *Pterodon emarginatus* fruits and characterise the volatile compounds. A 2^3^ full-factorial central composite design and response surface methodology were used to evaluate the effects of time (min), moisture (%) and microwave power (W) on the extraction yield. The process optimisation was based on the desirability function approach. The reaction time and moisture conditions were standardised in these analyses. The volatile oil composition was analysed by Gas Chromatography/Mass Spectrometry (GC/MS) in order to compare techniques extractions influences. Microwave irradiation showed excellent performance for extraction of the volatile oil from *Pterodon emarginatus* and there were some advantages in compare to conventional method with respect to the time (14 times), energy (6 times), reagents amounts and waste formation. About chemical composition presents significant differences with the type of extraction. Caryophyllene (25.65%) and *trans*-α-bisabolol (6.24%) were identified as major components in MAE sample while caryophyllene (6.75%) and γ-elemene (7.02%) are the components with higher relative percentage in CE samples. The microwaves assisted process shown an increase of economic interested compounds present in volatile oil.

## 1. Introduction

The Brazilian Cerrado (savannah) comprises hundreds of plant species that show potential use and feasibility for economic and therapeutic exploitation. Several of these species are used by local populations to treat various diseases that affect humans and animals. One of these medicinal plants is *Pterodon emarginatus* (Fabaceae), also called ‘‘sucupira’’ or ‘‘faveiro’’ that is distributed throughout central Brazil, in the states of Goiás, Minas Gerais and São Paulo.

The Fabaceae or Leguminosae family belongs to the order Fabales, which comprises about 650 genera and 18,000 species worldwide [1,2,3]. Approximately 200 general and 1,500 Fabaceae species are found in Brazil, with a particularly high representation from the Brazilian Cerrado, which contains around 777 species, distributed across approximately 101 genera [4,5]. The Fabaceae family anatomically covers three subfamilies, namely the Papilionoideae (Faboideae), Caesalpinoideae and Mimosoideae [6]. The seed oil and extracts of the bark and stems of *P. emarginatus* are used in Brazilian folk medicine to treat rheumatism, control diabetes, antimicrobial, anti-inflammatory and analgesic agent [7]. Among others, lupeol, botulin triterpenes, flavonoids, saponosides were identified in stem bark and seed extracts [8,9,10].

The fruit of *P. emarginatus* are used for the treatment of muscle aches, sprains, arthritis and arthrosis, anti-inflammatory and analgesic activity [11]. Studies of a crude extract of *P. emarginatus* fruits found an anti-inflammatory activity attributed to the presence of terpene compounds [12]. Also, the crude hexane extract of *P. emarginatus* fruit showed to protect against the oxidative and nitrosative stress induced by acute exercise in rats [13]. Characterisation of the hexane extract identified fatty acids, sesquiterpenes (α-caryophyllene, β-caryophyllene, myrcene, α-pinene, farnesene) and tricyclic furanoditerpenes, with the isolation of 6α, 7β-diacetoxy-vouacapan-17β-oate [14].

In this context, a recent technology namely microwave-assisted extraction (MAE) has been broadly used to extract various natural components from plants [15,16,17,18,19,20]. The MAE for the extraction of oils from natural substrates has been established as an alternative to conventional heating because it allows the decrease of the extraction time, the decrease of the volume solvent and the decrease of the amount of biomass needed by increasing the extraction yield [21,22,23]. The superior performance of microwave irradiation in these extraction processes could be related with the interaction of irradiation and water, microwave releases the essential oil and in situ water is transferred from the inside to the outside of the plant material [24]. The extraction efficiency can be affected by several properties such as the characteristics of the plant material, power level, duration of microwave irradiation, type and volume of solvent used, the ratio biomass/solvent, the size of the coupled distillation system and the vial format used for the extraction therefore the rational optimization of the microwave-assisted extraction is essential to achieve higher efficiencies. Considering the various biological activities associated with *P. emarginatus* constituents and other botanical species, the design of green and sustainable extraction methods and characterization of the essential oils derived from these plants is an important and trending area of research aimed at sourcing natural bioactive compounds. Here we report the optimisation of the microwave assisted extraction (MAE) with experimental design of volatile oil from the fruit of *P. emarginatus* considering the extraction time, power and properties of the plant material (humidity) and analyzed the chemical composition of the extract.

## 2. Results

### 2.1. Optimisation of Microwave-Assisted Extraction of Volatile Oil

Microwave-assisted extraction were performed using 30 g of dried fruits of *P. emargnatus* in 13.2 mL of water. To optimize the influence of experimental variables: the extraction time, moisture and microwave irradiation in the extraction yield we performed a 2^3^ experimental design. Table 1 shows the coded (in parentheses) and the decoded values of the independent variables, namely, time (min), moisture (water added to sample) (%) and microwave power (W), on the experimental extraction yields (% *v*/*w*) and volume (mL) of volatile oil obtained.

The association between the dependent and independent variables were described by fitting Equation (1) to the experimental data by multiple regression analysis. Analysis of variance (ANOVA) was then used to evaluate the fit of the model. The results showed the terms of the model that were statistically significant with confidence level of 95% (*p* < 0.05) and those that were not statistically significant. The ANOVA results for all responses are listed in Table 2.

Table 2 results indicate that the variables with the largest effects on extraction yield were the linear terms, time (X_1_) and power (X_3_), followed by the quadratic term, time (X_1_^2^) and the interaction between moisture and power (X_2_.X_3_). The linear term, moisture (X_2_), and the interaction between the time and power (X_1_.X_3_) were also significant (*p* < 0.05). The ANOVA showed that lack-of-fit was not significant for the model at 95% confidence interval (CI) (*p* > 0.05), confirming that the model satisfactorily represented the data. Also, the *R*^2^_adj_ was 0.9876, verifying that the fitness of the model to the extraction yield explained more than 98% of the total variability within the range of values studied. The model equation for extraction yield, without the insignificant terms, is given in Equation (1):

Extraction yield (%) = 0.012X_1_^2^ + 0.794X_2_ + 0.200X_3_ − 0.442X_1_ – 0.003X_2_X_3_ – 0.003X_2_X_3_ – 0.0006X_1_X_3_ − 42.49 (1)

**Figure 1:**
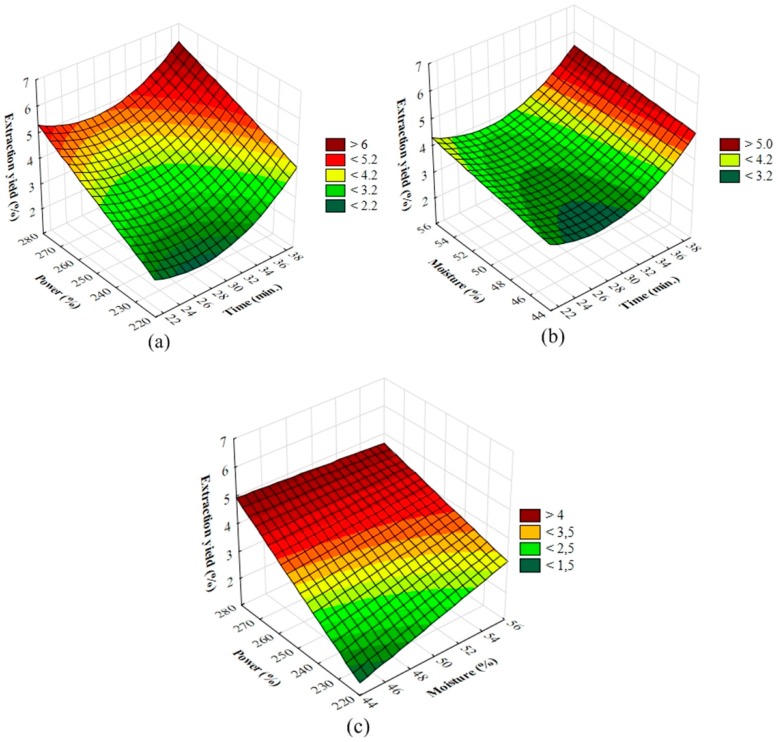
Response surface plots show the effect of time (min), moisture (%) and microwave power (W) on the extraction yield (%) (**a**) the yield values increased as time and power increased; (**b**) the yield values increased the extraction time in combination with moisture; (**c**) The yield values increased when low moisture and high power were tested.

The three-dimensional response surface plots were generated based on Equation (2), by fixing one independent variable at the zero level while the others were varied within the range studied, to analyse the effects of independent variables on the response (Figure 1). As shown in Figure 1a, the yield values increased as time and power increased. Also, the response surface plot of yield as a function of the time and moisture, which maintained the power fixed at the centre point value (Figure 1b), showed that increasing the extraction time in combination with moisture, resulted in increased yield. Alternatively, (Figure 1c) demonstrates that the use of low moisture and high power also led to an increase in extraction yield.

The contour and response surface model described by Equation (3) showed that increasing the extraction time and irradiation power resulted in higher yields and volume of extracted oil. The increase in moisture negatively influenced the extraction process.

**Figure 2:**
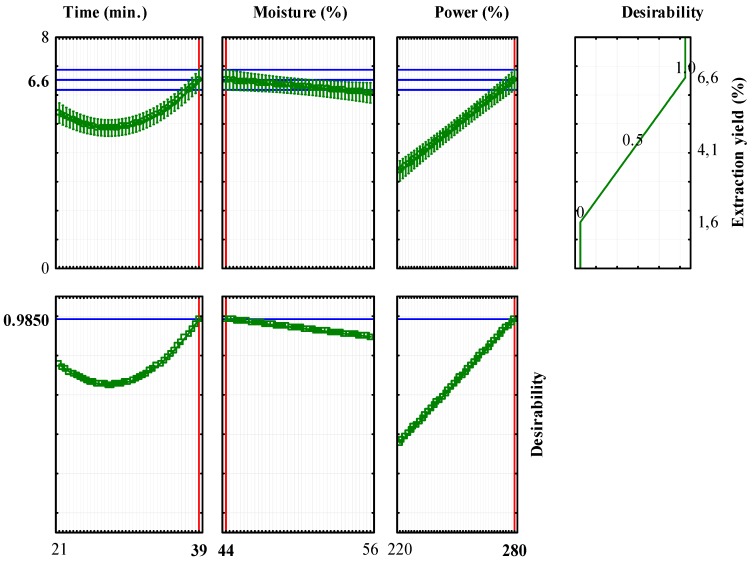
Profiles of predicted response values and desirability function. The red lines indicate experimental values after optimisation.

The desirability function was used to determine the optimum setting of the reaction conditions that led to the highest response level [25]. The optimisation algorithm allowed the elaboration of the profiles for predicted response values and desirability functions (Figure 2). The red vertical lines shown in the upper three rows of curves in Figure 2 indicate the maximal individual desirability relative to extraction yield, while those in the bottom row show the maximum global desirability.

The maximum global desirability, D = 0.9850, is reached when the optimised reaction conditions are implemented, i.e., 39 min extraction time, a low moisture of 44% and high power at 280 W. Therefore, it is expectable that using these experimental conditions an extraction yield of 6.6% *v*/*w* will be obtained.

### 2.2. Volatile Oil Extraction

The microwave-assisted extraction of volatile oil of 30 g of *P. emarginatus* fruits in 13.2 mL of water under the optimal established conditions, 39 min, 44% humidity and 280 W, yield 1.73 mL of the volatile oil, 5.76% (*v*/*w*). Conventional extraction was performed using the same reaction conditions as used in MAE (X_1_ = 39 min, X_2_ = 15 mL; X_3_ = 280 W) but without the microwave irradiation 0.44% (*v*/*w*). The conventional extraction using 50 g of biomass of dried crushed fruit of *P. emarginatus* in 500 mL of distilled water during 4 h yield 3.60 (% *v*/*w*) of volatile oil. The results (Table 3) confirm the efficacy of MAE to extract volatile oil from *P. emarginatus* fruits. Optimize MAE yielded more than ten times the quantity of oil that conventional extraction (CE) (5.76 versus 0.44% *v*/*w*) in 39 min and almost 2 times more oil than CE in 4 h.

Beyond the increase of the extraction yield the optimization of MEA of volatile oil of from *P. emarginatus* fruits led to the reduction of the quantity of solvent and the reduction of the extraction time. The extraction under conventional conditions requires 10 mL of water per gram of biomass while using the optimize conditions for MAE only 0.4 mL are needed. The reduction of the extraction time results in the reduction of the energy input. Under conventional heating were consumed 2.6 kW/h to obtained 1.73 mL of volatile oil from 50 g biomass and under microwave irradiation were consumed 0.1 kW/h per 30 g of biomass to obtained 1.80 mL of volatile oil. Therefore, using the optimized MAE the energy input was reduced from 5.33 to 0.11 kJ/g. The reduction of the extraction time and the energy input approach the method of the sustainability requirements that is also illustrated by the increase of the analytical Eco-scale from 86 to 89 being closer of 100, to the optimal value for green analysis [26] (Table 4).

### 2.3. Analysis of the Chemical Composition by Gas Chromatography Mass Spectrometry (GC/MS)

Chemical composition is an intrinsic property of the plant influenced by genetics, ontogeny, and environmental stimuli, such as the incidence of ultraviolet rays, amount of water, and soil nutrition. Also, there may still be variation between individuals of the same species, including geographically similar location [27].

Biomass treatment and the extraction method also could influence the chemical constitution of the sample. During the distillation process for example, water, acidity and temperature can cause the hydrolysis of esters, rearrangements, isomerizations, racemizations and oxidations [28].

The relative percentages of the components identified by GC/MS in the essential oil extract from fruits of *P. emarginatus* are presented in Table 5.

The GC/MS analysis was determined on the volatile oil obtained under the optimised oil extraction conditions and under conventional conditions. All the constituents identified were sesquiterpenes hydrocarbons. The main component of the volatile oil is γ-muurolene with 48.03 and 47.31% in MAE volatile oil and CE, respectively. However, the chemical composition presents significant differences with the type of extraction. Caryophyllene (25.65%) and *trans*-α-bisabolol (6.24%) were identified as major components in MAE sample while caryophyllene (6.75%) and γ-elemene (7.02%) are the components with higher relative percentage and *trans*-α-bisabolol is not detectable in CE samples (Table 5). This increases the chance of obtaining compounds with pharmacological and cosmetic potential. Follow bellow the major compounds chemical properties.

## 3. Discussion

*P. emarginatus* plays a vital role in the socio-economic development of the local region, and its essential oil is widely used in Brazilian folk medicine [10]. The data obtained in this study revealed a pronounced increase in the yield of essential oil from *P. emarginatus* fruits by MAE compared to CE. Nevertheless, the extraction itself derived from the experimental design had variation in yield results, which can be related not only to the amount of biomass and the extraction time but the way of pressing the fruits. For this work we used knives mill crusher type to obtain fruit powder sample. The more biomass is formed more efficient will be the volatile oil yield extraction. This is also true for the method by microwave irradiation [20].

This study used a fully adapted microwave distillation device intended only for extractions by Clevenger apparatus. This proof-of-principle study was demonstrated using a small quantity of plant material. Therefore, further tests should investigate the relative efficiency of this MAE approach using a greater amount of biomass than used in the present study.

The use of this technique is an excellent alternative for better utilization and efficiency of oil extraction of this species because it is a native species of Brazilian Savannah and still difficult to grow [10,15].

In the analysis of the chemical composition of the samples obtained by MAE and by CE it is noticed that the MAE was able to increase the obtaining of the majorities such as cariophyllene, γ-muuruleno and γ-elemeno. This is important because these compounds have biological properties such as anti-inflammatory, antimicrobial and antimicrobial properties that are of interest to the medical and cosmetic pharmaceutical field [12]. The qualitative and quantitative analysis of the essential oil from the fruits of *P. emarginatus* obtained under conventional conditions showed the presence of α-pinene, myrcene, caryophyllene and methyl, ethyl, and geraniol eugenol derivatives [27]. The compounds β-ilangeno, α-copaeene, β-caryophyllene, humulene, α-, γ- and δ-elemene, and cadinene were also identified [10,14].

Also, future studies that identify the bioactive properties of the main constituents separately from the essential oil and their mechanism of action are encouraged. MAE can be considered a suitable replacement for CE, requiring less biomass for higher essential oil yield, significantly less extraction time, energy use, CO_2_ emissions and solvent, highlighting its efficiency and bio-sustainability [21]. Furthermore, the MAE technique can be used to obtain secondary metabolites from other medicinal plants and, therefore, represents an important biotechnological advancement. The variables of the equipment (power, temperature, pressure and time), offer a range of possibilities that should vary per the extraction kinetics of each biomass. Finally, the characteristic of studies of microwave-assisted extraction, which stands out in technology is the reduction in the duration in time (14 times than conventional method), energy (six times than conventional method) and reagents/solvents.

## 4. Materials and Methods

### 4.1. Botanical Material

The fruits of *P. emarginatus* were collected from Jaraguá city in Goiás state, Brazil (16°53′21′′ S 49°15′12′′ W) and taxonomically identified by Prof. Dr. Josana Peixoto de Castro, State University of Goiás, Brazil. The voucher specimen (number 9324) was deposited in the Herbarium of the State University of Goiás, Brazil. The fruits were cleaned with flux of air and ground by knives crusher. The samples were maintained at room temperature (maximum 24 °C) until the analysis.

### 4.2. Conventional Extraction (CE)

The volatile oil was extracted by steam distillation in a closed flask system coupled to Clevenger apparatus [29]. Dried crushed fruit of *P. emarginatus* (50 g) was added into 500 mL of distilled water and the extraction cycle kept for 4 h.

### 4.3. Microwave-assisted Extraction, Experimental Design (MAE) and Statistical Analysis

The volatile oil was extracted by steam distillation in a closed flask system coupled to Clevenger apparatus and adapted to the reactor’s microwave apparatus (model SP, CEM Corporation, Matthews, NC, USA) following literature protocols [30]. The optimal conditions to perform MAE were established through experimental design.

A 2^3^ full-factorial design with center point was used to evaluate the main effects and interactions of the following variables: time (min), moisture (%) and microwave power (W) on response variable extraction yield (%). In this case, the percentage of water added in the powder material for the extraction, followed the amount determined by the experimental design. The choice of the parameters and their levels was based on the previous experimental studies. A total number of 13 runs of experiment including eight experiments of 2^3^ full-factorial design and five replicates at the center point were carried out. The independent variables and their levels are shown in Table 6. All the experiments were carried out at random, in order to minimize the effect of unexplained variability in the observed responses due to systematic errors.

The experimental data collected from the 2^3^ full-factorial with central composite design, were fitted to a polynomial function. Multiple regression analysis was used to fit Equation (2) to the experimental data by the least squares method.

The mathematical model was used to predict the response obtained from the experimental design applied:

(2) $$Y=b_{0}+\sum_{i=1}^{k}b_{i}X_{i}+\sum_{i=1}^{k}b_{\mathrm{ii}}X_{i}^{2}+\sum_{i<j}^{k}b_{\mathrm{ij}}X_{i}X_{j}+\varepsilon$$

where Y represents the predicted response, b_0_, is the model intercept, b_i_, b_ii_, and b_ij_ are the coefficients of the linear terms X_i_, X_i_^2^ and X_j_ are independent variables and ε corresponds to the model residue. The statistical significance of each coefficient term was determined by evaluating the *p*- and *F*-values with 95% confidence level (CL) obtained from the analysis of variance (ANOVA). The lack-of-fit of the regression model was evaluated with 95% CL. The statistical fit of the experimental results to the polynomial model equation was expressed by the coefficient of determination (*R*^2^) and the adjusted coefficient of determination (*R*^2^_adj_). Response surface plots were obtained by using the fitted model and by keeping one independent variable constant at zero level while varying the remaining two variables. Once an adjusted response to the polynomial model was obtained, the best conditions of volatile oil extraction were defined using desirability function analysis. All calculations and graphs were obtained using Statsoft version 7.0 (Statistica, TIBCO Software Inc., Palo Alto, CA, USA).

### 4.4. Yield of Volatile Oil

The yield of the volatile oil extracted from the fruit (% *v*/*w*) was calculated by directly measuring the volume (mL) of fluid on the collector tube Clevenger apparatus, and correlated to the amount of moisture-free sample, according to the protocols in literature [31].

### 4.5. Characterisation of the Chemical Composition of Volatile Oils by Gas Chromatography/Mass Spectrometry (GC/MS)

GC/MS was accomplished by a model GC/MS-QP2010 instrument (Shimadzu, Tokyo, Japan), equipped with an RTX-5MS column (30 m × 0.25 mm internal diameter). The carrier gas was helium, the flow rate was 1 mL/min, the injection volume was 1.0 µL, and the injector temperature was set at 200 °C. The temperature was initially increased at 3 °C/min until it reached 240 °C, then at 10 °C/min to 280 °C, where it was maintained for 10 min. The volatile oil constituents were identified by reference to the NIST 11 Mass Spectral Library (NIST11/2011/EPA/NIH) and by comparing their retention indices (RIs) with authentic mass spectra [32]. The RIs were calculated by co-injection with a mixture of C_8__C_32_ hydrocarbons (Sigma-Aldrich, St Louis, MO, USA) and using the Van den Dool and Kratz equation [32]. The relative percentage of the compounds within the volatile essential oil were obtained by area normalisation.

### 4.6. Calculation of Energy Consumption

The energy consumption was calculated using Equation (3) [33]:

*Energy consumption* = *P* × *t* (3)

where, *P* = equipment power (W) and *t* = time (min or h).

The hot plate (Fisatom model: 752A, Fisatom, São Paulo, SP, Brazil) and microwave heating (model SP, CEM Corporation, Matthews, NC, USA) used during the studies has power of 650 W and 280 W, respectively. These powers reported were used as standard to calculate the energy consumption.

### 4.7. Calculation of Analytical Eco-Scale

The sum of penalty points for the whole procedure should be included in the Eco-Scale calculation, according to the following formula [26], Analytical Eco-Scale = 100 − (total penalty points). The calculation result is ranked on a scale where a score >75 represents excellent green analysis, >50 represents acceptable green analysis, and <50 represents inadequate green analysis [26]. The basis for our concept of an analytical eco-scale is that the ideal green analysis has a value of 100. Recently, in organic synthesis the concept of eco-scale values has emerged as more accurate tool for evaluating the “ecofriendliness” of a given process, estimating the quality of the organic preparation based on yield, cost, safety, conditions, and ease of workup/purification. This can also be applied to the natural product field.

## 5. Conclusions

The microwave assisted-extraction has been shown to be feasible with particular interest in avoiding the need for organic solvents in volatile oil extraction from plants. In this work we proof that a minimum amount of water could be enough to bring good result in extraction. This green methodology appears to be an excellent alternative for obtaining terpenes from aromatic plants for academic purposes, as well as pharmaceutical, cosmetic and food fields.

## Figures and Tables

**Table 1:** Optimisation experimental results of the extraction process yield and volume of volatile oil obtained from *P. emarginatus* fruits.

Run	Time (min)(X_1_)	Moisture (%)(X_2_)	Power (W)(X_3_)	Yield (% *v*/*w*)	Vol. (mL)
1	21(−1)	44(−1)	220(−1)	1.6	0.5
2	21(−1)	44(−1)	280(+1)	5.3	1.6
3	21(−1)	56(+1)	220(−1)	3.3	1.0
4	21(−1)	56(+1)	280(+1)	5.3	1.6
5	39(+1)	44 (−1)	220(−1)	3.3	1.0
6	39(+1)	44 (−1)	280(+1)	6.6	2.0
7	39(+1)	56(+1)	220(−1)	5.0	1.5
8	39(+1)	56(+1)	280(+1)	6.0	1.8
9	30(0)	50(0)	250(0)	3.6	1.1
10	30(0)	50(0)	250(0)	3.6	1.1
11	30(0)	50(0)	250(0)	3.3	1.0
12	30(0)	50(0)	250(0)	3.6	1.1
13	30(0)	50(0)	250(0)	3.6	1.1

**Table 2:** Analysis of variance (ANOVA) results.

Source of Variation ^a^	Sum of Square	df	Mean Square	*F*-Value	*p*-Value
X_1_	3.65	1	3.65	202.50	0.00014 ^b^
X_1_^2^	3.14	1	3.14	174.38	0.00019 ^b^
X_2_	0.98	1	0.98	54.44	0.00180 ^b^
X_3_	12.50	1	12.50	694.44	0.00001 ^b^
X_1_X_2_	0.04	1	0.04	2.50	0.18900 ^d^
X_1_X_3_	0.25	1	0.25	13.61	0.02103 ^c^
X_2_X_3_	2.00	1	2.00	111.11	0.00046 ^b^
Lack of fit	0.04	1	0.04	2.50	0.18900 ^d^
Pure error	0.07	4	0.02		
Total sum of squares	22.67	12			
*R^2^*_adj_	0.9876				

^a^ X_1_ = Time (min.); X_2_ = Moisture (%); X_3_ = Power (W); ^b^ Significant at *p* < 0.01; ^c^ Significant at *p* < 0.05; ^d^ Not significant.

**Table 3:** Yield of *P. emarginatus* volatile oil by microwave-assisted extraction (MAE) compared to conventional extraction (CE).

Extraction	Medium Volume and Medium Yield	
	Volume (mL)	Yield (% *v*/*w*)
MAE	1.73	5.76
CE*	1.80	3.60
CE**	0.22	0.44

* conventional extraction during 4 h; ** conventional extraction during 39 min.

**Table 4:** Penalty points (PPs) for conventional extraction method versus microwave extraction method analysed by Gas Chromatography Mass Spectrometry (GC/MS).

Methods	Conventional	PPs	Microwave	PPs
Reagents	Biomass Sample: 50 g	2	Biomass Sample: 30 g	2
	H_2_O: 500 mL	3	H_2_O: 13.2 mL	2
Instruments	Hot plate (2.6 kW/h)	2	Microwave	0
	GC/MS	2	GC/MS	2
	Occupation hazard	0	Occupation hazard	0
	Waste	5	Waste	5
Total Penalty Points		14		11
Analytical Eco-Scale Score		86		89

**Table 5:** Chemical composition and respective percentages of the main constituents in the volatile oil obtained by microwave-assisted extraction (MAE) and conventional extraction (CE) of *P. emarginatus* fruits.

RI	Compound	MAE (%)	CE (%)	Chemical Properties
1436	γ-Elemene 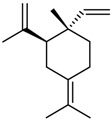	1.16	7.02	Molecular formula C_15_H_24_ CAS 5951-67-7 Molecular weight 204.352 Boiling point 125 °C at 8 mmHg Relative density 0.8782 at 20 °C Refraction index 1.5130 at 26 °C Soluble in benzene and acetone
1376	α-Copaene 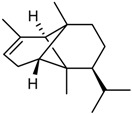	3.54	1.05	Molecular formula C_15_H_24_ CAS-3856-25-5 Molecular weight 204.352 Boiling point 248 °C at 760 mmHg and 124 °C at 15 mmHg Relative density 0.8996 at 20 °C Refraction index 1.4894 at 20 °C Soluble in ethanol acetone, acetic acid e ligroin
1494	*E*-Caryophyllene 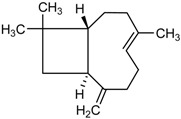	25.65	6.75	Molecular formula C_15_H_24_ CAS 87-44-5 Molecular weight 204.352 Boiling point 262–264 °C at 760 mmHg, 122 °C at 13.5 mmHg and 118–119 °C at 9.7 mmHg Relative density 0.9075 at 20 °C Refraction index 1.4986 at 20 °C Very soluble in benzene
1454	α-Humulene 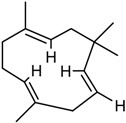	4.92	2.49	Molecular formula C_15_H_24_ CAS 6753-98- 6Molecular weight 204.352 Boiling point 166–168 °C at 760 mmHg and 123 °C at 10 mmHg Relative density 0.8905 at 20 °C Refraction index 1.5038 at 20 °C
1531	*trans*-α-Bisabolol 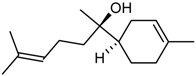	6.24	--	Molecular formula C_15_H_24_ CAS 17627-44-0 Molecular weight 222.36 Boiling point 153 °C at 12 mmHg mmHg and 123 °C at 10 mmHg Relative density 0.9211 at 20 °C Refraction index 1.493–1.497 at 20 °C Soluble in ethanol, isopropanol and parafin
1469	allo-Aromadendrene 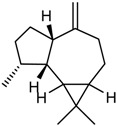	1.31	0.92	Molecular formula C_15_H_24_ CAS 25246-27-9 Molecular weight 222.36 Boiling point 265–267 °C at 760 mmHg Relative density 0.923 at 20 °C Refraction index 1.501 at 20 °C
1523	*cis*-Sesquisabinene 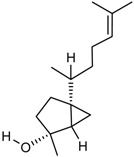	1.23	0.70	Molecular formula C_15_H_24_ Molecular weight 204.352 Boiling point 286 °C at 760 mmHg (predictive value).
1479	γ-Muurolene 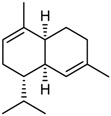	**48.03**	**47.31**	Molecular formula C_15_H_24_ Molecular weight 204.352 Boiling point 126 °C at 12 mmHg Relative density 0.9182 at 20 °C Refraction index 1.3166 at 20 °C

**Table 6:** Uncoded and coded levels of the independent variables used to extract the volatile oil from *P. emarginatus* fruits.

Variables	Levels		
	−1	0	1
X_1_	21	30	39
X_2_	44	50	56
X_3_	220	250	280

X_1_ = extraction time (min); X_2_ = moisture (%); X_3_ = power microwave irradiation (W).

## References

[1] Polhill R.M., Raven P.H. (1981). Advances in Legume Systematics.

[2] Judd W.S., Campbell C.S., Kellogg E.A., Stevens P.F. (1999). Plant Systematics: A Phylogenetic Approach.

[3] Souza V.C., Lorenzi H. (2005). Botânica Sistemática: Guia Ilustrado para Identificação das Famílias de Angiospermas da Flora Brasileira, Baseado em APG II.

[4] Ratter J.A., Ribeiro J.F., Briggewater S. (1997). The Brazilian Cerrado vegetation and threats to its biodiversity. Ann. Bot..

[5] Mendonça R.C., Felfili J.M., Walter B.M.T., Silva M.C., Rezende A.V., Filgueiras A.V., Nogueira P.E., Sano S.M., de Almeida S.P. (1998). Flora Vascular do Cerrado. Cerrado: Ambiente e Flora.

[6] Solereder H. (1908). Systematic Anatomy of the Dicotyledons: A Handbook for Laboratories of Pure and Applied Botany.

[7] Almeida M.E., Gottlieb O.R. (1975). Further isoflavones from *Pterodon apparicioi*. Phytochemistry.

[8] Moraes W.F. (2007). Estudo Fitoquímico e Avaliação das Atividades Analgésica e Anti-Inflamatória do Extrato Etanólico, Frações e Substância Isolada da Casca do Caule de Pterodon Emarginatus Vog. (Sucupira). Master’s Thesis.

[9] Bustamante K.G.L., Figueiredo A.D.L., Soares M.L., Bara M.T.F., Ferreira H.D., Rezende M.H., Pimenta F.C., Paula J.R. (2010). Avaliação da atividade antimicrobiana do extrato etanólico bruto da casca da sucupira branca (*Pterodon emarginatus* Vogel)—Fabaceae. Rev. Bras. Plant. Med..

[10] Santos A.P., Zatta D.T., Moraes W.F., Bara M.T., Ferri P., Silva M.R., Paula J.R. (2010). Composição química, atividade antimicrobiana do óleo essencial e ocorrência de esteróides nas folhas de *Pterodon emarginatus* Vogel, Fabaceae. Rev. Bras. Farmacogn..

[11] Mors W.B., Santos M.F., Monteiro H.J., Gilbert B., Pellegrino J. (1967). Chemoprophylactic agent in schistosomiasis: 14, 15-epoxygeranylgeraniol. Science.

[12] Carvalho J.C.T., Sertié J.A.A., Barbosa M.V.J., Patricio K.C.M., Caputo L.R.G., Sarti S.J., Ferreira L.P., Bastos J.K. (1999). Anti-inflamatory activity of the crude extract from the fruits of *Pterodon emarginatus* Vog. J. Ethnopharmacol..

[13] Paula F.B.A., Gouvêa C.M.C.P., Alfredo P.P., Salgado I. (2005). Protective action of a hexane crude extract of *Pterodon emarginatus* fruits against oxidative and nitrosative stress induced by acute exercise in rats. BMC Complement. Altern. Med..

[14] Teixeira D.F. (2003). Estudo Químico e Avaliação Biológica de Attalea 11xcels Mart. Ex Spreng. (Urucuri) e Pterodon Emarginatus Vog. (Sucupira-Branca) em Aedes aegypti. Master’s Thesis.

[15] Ganzler K., Bati J., Valko K. (1986). A new method for the extraction and high-performance liquid chromatographic determination of vicine and convicine in faba beans. Chromatography.

[16] Campos A.M., Craveiro A.A., Teixeira T.C. (1990). Óleo Essencial das Sementes de *Pterodon polygalaeflorus* Benth. Resumos da 13^a^ Reunião da Sociedade Brasileira de Química.

[17] Collin G.J., Lord D., Allaire J., Gagnon D. (1991). Essential oil and microwave extracts. Parfums Cosmèt. Aromes.

[18] Chen S.S., Spiro M. (1994). Study of microwave extraction of essential oil constituents from plant materials. J. Microw. Power Electromagn. Energy.

[19] Lucchesi M.E., Chemat F., Smadja J. (2004). Solvent-free microwave extraction: An innovative tool for rapid extraction of essential oil from aromatic herbs and spices. J. Microw. Power Electromagn. Energy.

[20] Wang H.W., Liu Y.Q., Wei S.L., Yan Z.J., Lu K. (2010). Comparison of microwave-assisted and conventional hydrodistillation in the extraction of essential oils from mango (*Mangifera indica* L.) flowers. Molecules.

[21] Kahriman N., Yayh B., Yücel M., Karaoglu S.A., Yayh N. (2012). Chemical constituents and antimicrobial activity of the essential oil from *Vicia dadianorum* extracted by hydro and microwave distillations. Rec. Nat. Prod..

[22] Phutdhawong W., Kawaree R., Sanjaiya S., Sengpracha W., Buddhasukh D. (2007). Microwave-assisted isolation of essential oil of *Cinnamomum iners* Reinw. ex Bl.: Comparison with conventional hydrodistillation. Molecules.

[23] Abraham W.R. (2001). Bioactive sesquiterpenes produced by fungi are they useful for humans as well?. Curr. Med. Chem..

[24] Vian M.A., Fernandez X., Visinoni F., Chemat F. (2008). Microwave hydrodiffusion and gravity, a new technique for extraction of essential oils. J. Chromatogr. A..

[25] Wei L., Zhang Y., Jiang B. (2013). Comparison of microwave-assisted hydrodistillation with the traditional hydrodistillation method in the extraction of essential oils from dwarfed *Cinnamomum camphora* var. *Linaolifera fujita* leaves and twigs. Adv. J. Food Sci. Technol..

[26] Gobbo-Neto L., Lopes N.P. (2007). Plantas medicinais: Fatores de influência no conteúdo de metabólitos secundários. Quím. Nova.

[27] Santos A.S., Alves S.D.M., Figueirêdo F.J.C., da Rocha Neto O.G. (2004). Descrição de Sistema e de Métodos de Extração de Óleos Essenciais e Determinação de Umidade de Biomassa em Laboratório, Embrapa Amazônia Oriental.

[28] Velasco C.A. (2007). Microwave Extraction of Peppermint Oil in Comparison to Current Practice Steam Extraction. Master’s Thesis.

[29] Figueirêdo F.J.C., Alves S.D.M., Santos A.S., da Rocha Neto O.G. (2004). Rendimento e Qualidade Físico-Química de Óleo Essencial Extraído de Diferentes Composições da Biomassa Área de Pimenta Longa.

[30] Adams R.P. (2007). Identification of Essential Oil Components by Gas Chromatography/Mass Spectroscopy.

[31] Riese J., Grunewald M., Lier S. (2014). Utilization of renewably generated power in the chemical process industry. Energ. Sustain. Soc..

[32] Van Den Doll H., Kratz P.D.J.A. (1963). Generalization of the retention index system including linear temperature programmed gas-liquid partition chromatography. J. Chromatogr..

[33] Gałuszka A., Konieczka P., Migaszewski Z.M., Namie’snik J. (2012). Analytical Eco-Scale for assessing the greenness of analytical procedures. Trends Anal. Chem..

